# Melatonin: A Versatile Protector against Oxidative DNA Damage

**DOI:** 10.3390/molecules23030530

**Published:** 2018-02-27

**Authors:** Annia Galano, Dun-Xian Tan, Russel J. Reiter

**Affiliations:** 1 Departamento de Química, Universidad Autónoma Metropolitana-Iztapalapa, San Rafael Atlixco 186, Col. Vicentina, Iztapalapa, C. P. 09340, Mexico D. F. Mexico; 2 Department of Cellular and Structural Biology, UT Health Science Center, San Antonio, TX 78229, USA; tan@uthscsa.edu (D.-X.T.); reiter@uthscsa.edu (R.J.R.)

**Keywords:** antioxidant activity, metal chelation, free radical scavenger, antioxidative protection, antioxidative mechanisms

## Abstract

Oxidative damage to DNA has important implications for human health and has been identified as a key factor in the onset and development of numerous diseases. Thus, it is evident that preventing DNA from oxidative damage is crucial for humans and for any living organism. Melatonin is an astonishingly versatile molecule in this context. It can offer both direct and indirect protection against a wide variety of damaging agents and through multiple pathways, which may (or may not) take place simultaneously. They include direct antioxidative protection, which is mediated by melatonin’s free radical scavenging activity, and also indirect ways of action. The latter include, at least: (i) inhibition of metal-induced DNA damage; (ii) protection against non-radical triggers of oxidative DNA damage; (iii) continuous protection after being metabolized; (iv) activation of antioxidative enzymes; (v) inhibition of pro-oxidative enzymes; and (vi) boosting of the DNA repair machinery. The rather unique capability of melatonin to exhibit multiple neutralizing actions against diverse threatening factors, together with its low toxicity and its ability to cross biological barriers, are all significant to its efficiency for preventing oxidative damage to DNA.

## 1. Introduction

Oxidative damage (OD) to DNA may compromise the genomic integrity [1] and, consequently, it has important implications for human health. In fact, OD has been identified as a key factor in the onset and development of numerous diseases. Thus, it is evident that preventing DNA from OD is crucial for humans and for any living organism. OD to DNA increases to unhealthy levels as a consequence of oxidative stress (OS), which is a chemical stress arising from the imbalance between the production and consumption of oxidants. Among such oxidants, free radicals (FR) seem to be particularly relevant, and there is a wide variety of them in living systems. At the same time, there are multiple endogenous and exogenous factors that contribute to elevate the levels of FR. Some examples are ischemia, infections, physical or mental stress, aging, pollution, radiation, heavy alcohol consumption, cigarette smoke, the intake of certain drugs, etc [2,3,4,5,6,7,8,9,10,11,12,13,14,15]. It is then logical that antioxidants that are efficient FR scavengers are potential candidates to reduce OD to DNA. 

There is abundant evidence on the antioxidant protection exerted by melatonin (*N*-acetyl-5-methoxytryptamine) and related compounds [16,17,18,19,20,21,22,23,24,25,26,27,28]. Such protective effects can involve diverse routes including free radical scavenging [29,30,31,32,33,34,35,36,37], but also the deactivation of other oxidants [38,39,40,41,42] and the inhibition of metal-induced lipid peroxidation [43,44,45,46,47,48]. The data gathered so far on the antioxidant activity of melatonin is so convincing that it has led to the hypothesis that one of the main functions of melatonin in living organisms is to protect them from OD [49]. Indeed, it has been found that low levels of endogenous melatonin in humans may lead to high levels of oxidative DNA damage [50].

In addition, melatonin metabolites also have antioxidant effects, which maintains protection against oxidants after melatonin is metabolized [16,51,52]. This combined action has been proposed as one of the reason why melatonin is highly effective as an antioxidant and capable of providing long-term protection against OS [52]. It has also been proposed that melatonin and its metabolites work in a “task-division” way, with some of them acting mainly as FR scavengers, while others act as metal chelating agents and inhibitors of the hydroxyl radical (^•^OH) production [19]. 

Based on the above mentioned facts, melatonin protects DNA from oxidation through different mechanisms, including its chemical antioxidant effects. Here, the data published to date regarding this particular action, and other possible protective mechanisms, are reviewed. In addition, some relevant aspects of OD to DNA are also discussed.

## 2. Oxidative Damage to DNA 

### 2.1. Induction by Free Radicals.

Free radicals can damage DNA directly or indirectly [53,54]. The direct damage often involves hydrogen atom transfer (HAT), radical adduct formation (RAF), or single electron transfer (SET) routes. The indirect damage arises from the action of the electrophilic species yielded when FR react with biomolecules such as lipids, proteins, and other cellular components. For example, the peroxidation of polyunsaturated fatty acids produces a variety of aldehydes that can lead to the formation of DNA adducts [55]. 

Regarding direct routes, it has been proposed that the ^•^OH is responsible for the most common oxidative damage to DNA [56,57]. This is probably because ^•^OH is the most reactive and electrophilic of the reactive oxygen species (ROS) [58]. The ^•^OH can be produced by ultraviolet and ionizing radiations or from other radicals arising from enzymatic reactions. However, the main intracellular sources of ^•^OH probably are the Fenton reaction and the metal catalyzed Haber-Weiss recombination (MC-HWR). Although there are several metal ions that, because of their redox properties, may be involved in such processes, only the chemical reactions with iron and copper are used next to illustrate them. The Fenton reaction involves the reduced forms of the metal ions:

Fe(II) + H_2_O_2_ → Fe(III) +OH^−^ + ^•^OH

Cu(I) + H_2_O_2_ → Cu(II) +OH^−^ + ^•^OH

The first step of the MC-HWR is:

Fe(III) + O_2_^•−^ → Fe(II) + O_2_

Cu(II) + O_2_^•−^ → Cu(I) + O_2_,

while the second step corresponds to the Fenton reaction.

Since the metal oxidized forms, Fe(III) and Cu(II), are their most abundant and stable oxidative state, the first step of the MC-HWR is more important in biological systems than the direct Fenton reaction. In other words, the reduction process -Fe(III) to Fe(II) or Cu(II) to Cu(I)- is the crucial step to the ^•^OH production. Thus, if the formation of Fe(II) or Cu(I) is inhibited so is ^•^OH production through the Fenton reaction, and therefore the ^•^OH-related oxidative damage. This is an important aspect of the ^•^OH production via the MC-HWR, because chelating agents able of decreasing the viability of Fe(III) and Cu(II) reduction reactions are expected to be effective in preventing, or inhibiting, oxidative damage to DNA.

**Scheme 1:**
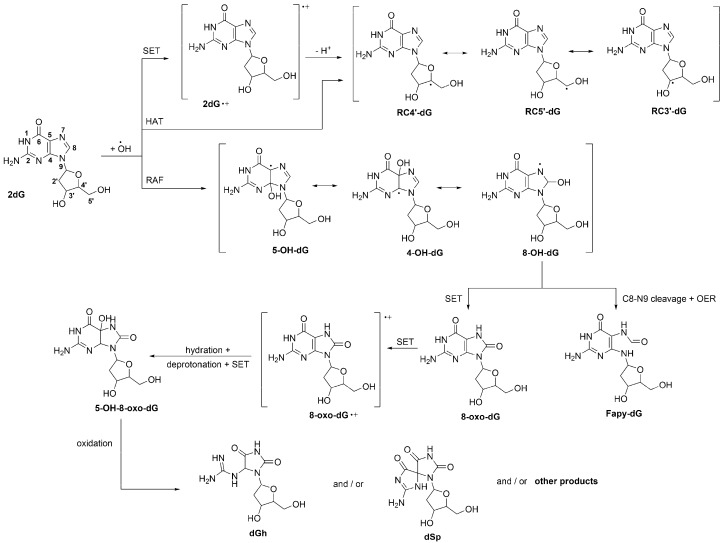
Site numbering of 2′-deoxyguanosine (2dG), and multiple pathways of its ^•^OH-initiated oxidation. OER = one-electron reduction.

From a chemical point of view, the direct reaction between ^•^OH and DNA is a multifaceted process. Due to the high reactivity of this radical, and its consequent low selectivity, such reaction can yield a myriad of products. One of the possible reaction channels is the SET, yielding a radical cation [59], which is expected to involve mainly guanosine sites. This is because guanosine is the most easily oxidized of the nucleic acid bases, with a reduction potential of 1.29 V vs NHE [60]. The fact that guanosine has the lowest ionization potential among the DNA components explains why it is the main sink for hole transfer in double-stranded DNA [61]. The radical cation generated by SET can readily deprotonate (Scheme 1), under physiological conditions, yielding carbon-centered radicals in the deoxyribose unit [62], which are involved in one of the most important types of DNA damage (strand scissions) [63,64]. Such carbon-centered radicals can also be directly produced by HAT from the sugar sites to ^•^OH [65,66,67]. Addition of this radical to 2′-deoxyguanosine (2dG) yields adducts on different C sites in the imidazole ring (Scheme 1) [57,68]. In turn, the 8-OH-dG adduct can further evolve. It may yield 8-oxo-7,8-dihydro-2′-deoxyguanosine (8-oxo-dG), due to one-electron oxidation, or 2,6-diamino-4-hydroxy-5-formamidopyrimidine 2′-deoxynucleoside (Fapy-dG) by cleavage of the C8-N9 bond plus one-electron reduction [53]. 

The 8-oxo-dG is one of the most abundant DNA lesions, and it considered as a biomarker of oxidative stress [69,70]. It has been estimated that up to 100,000 8-oxo-dG lesions can occur daily in DNA per cell [1,60]. The reduction potential of 8-oxo-dG is even lower (0.74 V vs. NHE) than that of guanosine [71]. Therefore, it can be further oxidized by a large variety of oxidants [72,73,74,75,76], yielding the corresponding radical cation, which can be transformed into the 5-hydroxy-substituted derivative of 8-oxo-dG (5-OH-8-oxo-dG) by hydration, deprotonation and another SET reaction (Scheme 1) [75,76]. This species can also be oxidized, yielding 2′-deoxynucleoside (dGh), spiroiminodihydantoin 2′-deoxynucleoside (dSp), and/or other products [75,76]. The relative abundance of such products would be influenced by the reacting species and by environmental factors. However, under physiological conditions dGh and dSp are both expected to be formed [53].

It seems worthwhile mentioning that, albeit they are not reviewed here, other nucleosides are also susceptible to be damaged by ^•^OH and other free radicals. The chemical routes involved in such processes are similar to those described here for 2dG [77,78,79]. In addition, oxidants such as FR, can produce not only single but multiple lesions to a DNA molecule, which may lead to the formation of cross-linked products [80,81,82,83,84,85,86].

### 2.2. Other Causes

In addition to the previously discussed metal-induced damage, which can be attributed at least partially to the production of ^•^OH radicals, there are other possible factors that may contribute to DNA lesions. Some halogen compounds such as HOCl and HOBr, which are produced as a response to inflammation, can cause structural modifications to DNA [87,88,89]. In fact, one of the products yielded by such processes, 5-chloro-2′-deoxycytidine, is considered a biomarker of chronic inflammation [90,91]. Nitric oxide (^•^NO) and superoxide radical anion (O_2_^−•^) are also produced as a consequence of immune responses to inflammation [92]. Albeit they do not directly damage DNA, their reaction yields peroxynitrite (ONOO^−^) [93], which reacts with DNA [94,95,96]. Guanine residues seems to be the main target of ONOO^−^, since its reaction with these residues is significantly faster than those involving other nucleosides, and its reactions with 8-oxo-dG is even more favorable than with the parent molecule [97]. 

Some by-products of lipid peroxidation also induce damaged to DNA. For example, aldehydes produced in this process can react with DNA, by RAF, yielding different products [55,98]. In turn, they can lead to DNA interstrand cross-links [99]. Other chemicals that can damage DNA are 17β-estradiol [100], 1-methyl-4-phenyl-1,2,3,6-tetrahydropyridine [101], d-aminolevulinic acid [102], thioacetamide [103], bisphenol A [104], methyl methanesulfonate [105] and the amyloid beta peptide [106]. In addition, there are other factors that represent a risk to DNA integrity such as radiation [107,108,109], ischemia/reperfusion [110], intracerebral hemorrhage [111] and aging [112].

### 2.3. Consequences for Human Health

**Figure 1:**
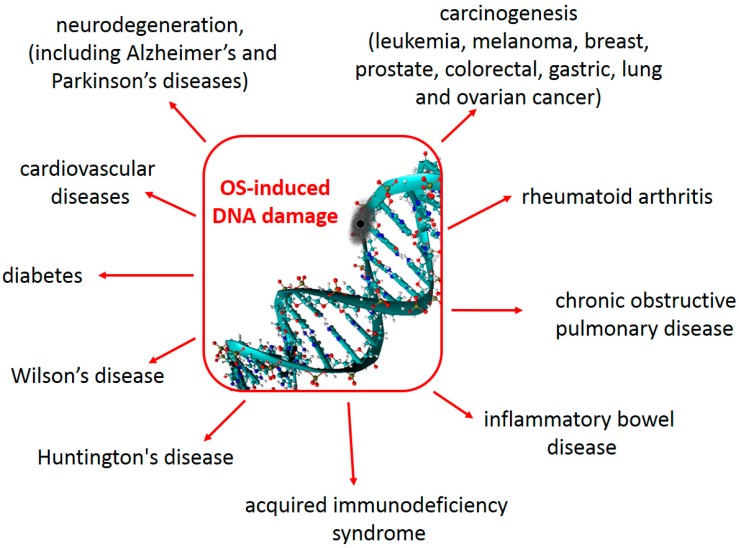
Some consequences to human health, derived from oxidative damage to DNA. See text for details and references.

DNA damage has serious, and sometimes life-threatening, consequences to human health (Figure 1). In fact the evidence gathered so far, in this regard, is so abundant that it is not possible to review it all here. Thus, only some will be mentioned as illustrative examples of the highly deleterious effects that structural modifications to DNA imposes to human health. In particular OS-induced DNA damage has been associated with a wide variety of diseases [113,114]. 

It has been proposed that this kind of damage may play a key role in carcinogenesis induced by chronic inflammation [115,116]. Elevated levels of OS and/or DNA damage have been found in cases of human breast, prostate, colorectal, gastric, lung and ovarian cancer; as well as in leukemia, melanoma and lymphoma patients [117]. The relationship between oxidative DNA damage and cancer arises, at least partially, from the fact that DNA lesions that are not detected during replication, such as 8-oxo-dG, can lead to mutations [118,119]. In addition, agents increasing oxidative DNA damage usually enhance the risk of cancer development, while diets rich in fruits and vegetables (which contain abundant antioxidants) decrease both oxidative DNA damage and cancer incidence [120]. Three main mechanisms have been proposed to contribute to DNA damage, by environmental agents, leading to carcinogenesis. They are: (i) oxidative DNA damage, involving the production of 8-oxo-dG; (ii) the formation of lipid peroxide-derived DNA adducts; and (iii) the methylation of cytosine by free radicals [121].

OS-induced DNA damage has also been identified as an important contributing factor to neurodegeneration. It initiates a series of events that promote neuronal loss, following central nervous system (CNS) injuries [122]. Increased nuclear and mitochondrial DNA oxidation have been observed in Alzheimer’s disease (AD), arising from the attack of reactive oxygen species (ROS) to DNA bases and from the impairment of DNA repair mechanisms [123]. Amyloid beta-induced oxidative DNA damage also contributes to the development and progression of this disease [124]. Elevated levels of mitochondrial DNA oxidation products have been found in cases of mild cognitive impairment and initial stages of AD, which has led to the suggestion that oxidative damage to DNA is an early event in AD [125]. Levels of 8-OH-dG in the cerebrospinal fluid (CSF), higher than in control groups, have been found not only in patients with AD but also in patients with Parkinson’s disease [126,127,128]. The same trend was found for sporadic amyotrophic lateral sclerosis, and the 8-OH-dG in the CSF was positively correlated with the illness duration [129]. However, in the particular case of PD, it has been proposed that while the levels of 8-OH-dG might be used as an “early-stage marker”, the disease progression might be characterized by a decrease of such levels in the CSF [130]. 

Oxidative DNA damage also seems to be involved in the development of cardiovascular diseases [131]. This kind of damage was found to be higher in coronary artery disease patients than in healthy subjects and to be potentiated by metabolic syndrome, which causes an increase in OS [132]. In addition continuous flow left ventricular assist devices implanted in heart failure patients were found to lead to elevated OS and DNA damage in blood leukocytes, and also to malfunction in DNA repair pathways [133]. The 8-OH-dG levels were found to be elevated in the serum and myocardium of patients with heart failure, compared to those in control subjects [134].

There are many other health disorders that have been related to high OS and OS-induced DNA damage. One of them is the Wilson’s disease, which is characterized by a copper accumulation, and the consequent increase in ROS production [135,136]. Others are inflammatory bowel disease (including both ulcerative colitis and Crohn’s disease) [137,138], diabetes [139] and its complications [140,141,142], acquired immunodeficiency syndrome [143,144], Huntington’s disease [145,146], rheumatoid arthritis [147,148,149], and chronic obstructive pulmonary disease [150], just to mention a few.

## 3. Antioxidant Protection 

Shielding biomolecules in general, and DNA in particular, from oxidative damage can be achieved in different ways that, depending on the moment at which they take place, might be roughly classified as prevention or repairing strategies [151]. Antioxidant prevention involves deactivating free radicals (or other oxidants) or inhibiting their formation, thus they do not reach biological targets. In addition, DNA repair should occur before replication to maintain genomic integrity and a healthy status. Enzymatic DNA repair plays an essential role in the defense mechanisms of living organisms, albeit in some particular cases it can also involve chemical pathways [152].

Melatonin is produced by the pineal gland, although it also is found in several extra-pineal organs [153,154,155,156,157,158]. It is best known for its regulatory role in circadian and seasonal rhythms [159,160,161]. However, there is increasing evidence on its many other biological functions [162]. Some examples are its anti-inflammatory and immune-enhancing properties [163,164,165], its homeostatic role in the mitochondrion [166,167,168] and in maintaining the fluidity of biological membranes [169]. In addition, melatonin has well-documented antioxidant capacity. As mentioned in the Introduction section, it has been proposed that one of the main functions of melatonin in living organisms is to protect them from OD [49]. Melatonin has also been classified as a mitochondria targeted antioxidant, acting as a “firewall” against FR [170]. We certainly agree with those statements. Since melatonin is a highly versatile molecule that plays diverse roles in living organisms, it might be difficult to establish (beyond any doubt) the relative importance of its many functions. However, its antioxidant protection is definitively a very important one.

**Figure 2:**
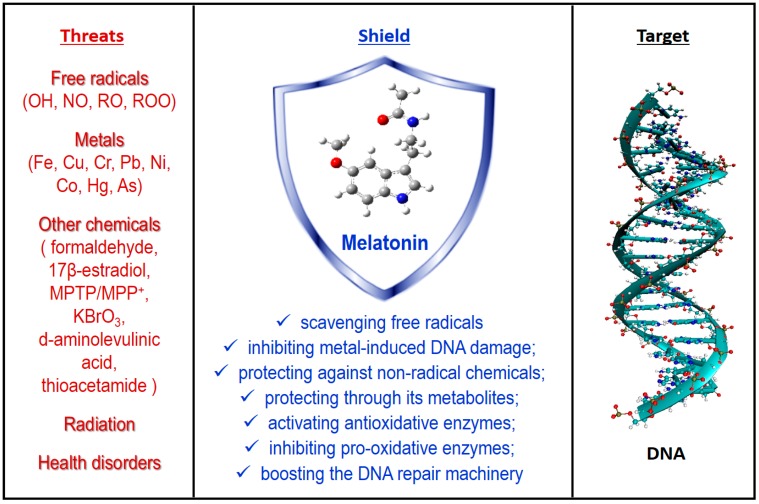
Some threats to DNA integrity and some pathways involved in melatonin’s protection. See text for details and references.

Melatonin has protective effects against OD to DNA, and also beneficial effects regarding many of the previously mentioned diseases. Since elevated ROS levels are among the major causes of DNA damage, the protection exerted by melatonin has been largely attributed to its antioxidant capacity (AOC) [171]. Melatonin’s AOC can be exerted not only directly, i.e., through its FR scavenging activity, but also indirectly (Figure 2), for example through its metabolites [172,173], by stimulation of antioxidative enzymes [15] or by modulating DNA repair pathways [171,174].

Some of the melatonin’s features make it particularly efficient for exerting AOC protection, since they are in line with those expected in ideal antioxidants [175]. One of them is its amphiphilicity, which allows melatonin to readily cross physiological barriers [176,177]. Therefore, it can provide on-site protection to DNA, against locally generated FR [171]. Another important feature of melatonin is its low toxicity, over a wide dose range. There is a large number of studies, in both animals and humans, indicating that short-term use of melatonin is safe, even in massive doses, while long-term administration may induce only mild (if any) adverse effects, comparable to placebo treatment [178]. The only significant short-term side effect reported after oral ingestion of ≤5 mg of melatonin by normal healthy adults was sleepiness [179]. Similar doses (administered as prolonged-release melatonin preparations) were found to be efficient and safe for the treatment of insomnia in children and adolescents with autism spectrum disorder [180]. Several in vivo studies on animals, involving high doses of melatonin showed that chances of acute and/or chronic toxicity of melatonin is extremely low [181,182,183,184]. In addition, oral doses of melatonin (up to 1 g daily), taken by human volunteers, resulted in no negative side effects [185]. In fact, there are no reports that exogenous melatonin causes any serious adverse effects. Thus, the general agreement that melatonin has minimal toxicity over a very wide dose range seems to be well justified. This allows considering supplementary intakes of this compound, to increase its level beyond those arising from endogenous production, thus boosting its capability to protect biomolecules in general, and DNA in particular, from OD. More information on the safety of melatonin and its clinical utility can be found elsewhere [186].

### 3.1. Free radical Scavenging Activity

Melatonin is capable of efficiently scavenging a wide variety of free radicals. Some of them are ^•^OH, alkoxy radicals (RO^•^), peroxy radicals (ROO^•^), and ^•^NO [32,33,37,187,188,189,190,191]. It also scavenges other, non-radical, oxidants such as hydrogen peroxide (H_2_O_2_) [192,193], singlet oxygen (^1^O_2_) [38] and ONOO^−^ [194]. The relationship between these actions, and the protection exerted by melatonin against oxidative damage to DNA has been well documented. 

**Scheme 2:**
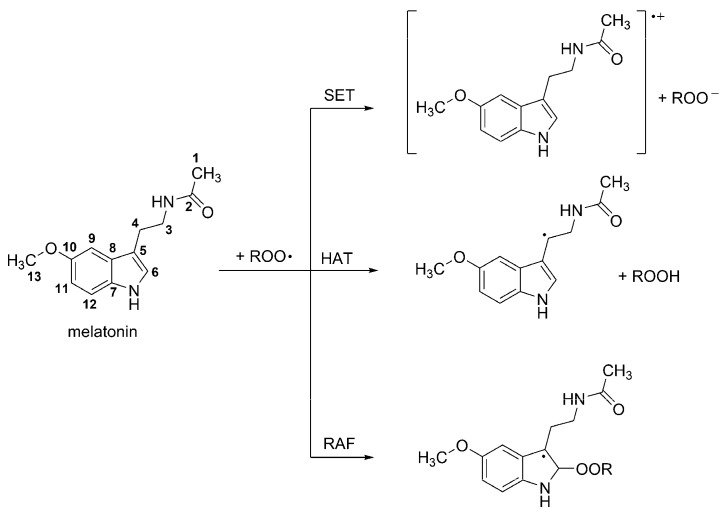
Site numbering of melatonin, and chemical pathways involved in its peroxyl radical scavenging activity.

Several reaction mechanisms have been investigated regarding the chemical protection exerted by melatonin through its free radical scavenging activity, including SET, HAT and RAF. It has been proposed that the relative importance of such mechanisms, as well as that of the different reaction sites, is influenced by the reacting FR. For example for FR of moderate reactivity, such as ROO^•^, the main reaction pathways are HAT from site 4 and RAF at site 6 [32] (Scheme 2). However when R has a high electrophilic character (for example CCl_3_OO^•^) the SET pathway contributes to a significant extent to the FR scavenging activity of melatonin. On the other hand, if the reacting FR is ^•^OH (which is more reactive than ROO^•^) the number of reaction paths involved in melatonin’s scavenging activity increases. In this case HAT occurs from sites C1, C3, C4, C6 and C7; and RAF at sites C5 to C12. Therefore, reaction with ^•^OH would yield a wide variety of products. 

In addition, melatonin derivatives have been designed to increase its antioxidative protection via SET [195,196,197,198,199,200,201,202]. Such a design was rationalized considering that the electron-rich aromatic ring promotes the redox behavior of melatonin, in particular as an electron donor. Therefore, introducing groups that stabilizes the indole ring, by increasing electron delocalization, might contribute to an improved antioxidant activity. Some of the melatonin derivatives designed that way resulted to be, in fact, better direct antioxidants than the parent molecule. 

The role of the FR scavenging activity of melatonin on its protective effects against OD to DNA has been well documented. Liang et al. [203] have recently shown that melatonin protects somatic cell nuclear transfer porcine embryos from OS-induced DNA damage. It was proposed that such protection involves the quenching of the FR arising from exposure to hydrogen peroxide (H_2_O_2_). In these experiments melatonin decreased the amounts of intracellular ROS, at the same time that it elevated the intracellular glutathione (GSH) levels, thus preventing H_2_O_2_-induced mitochondrial dysfunction. 

Erenberk et al. [204] investigated the effects of melatonin in preventing the DNA damage associated to the consumption of phenytoin sodium (PHT-Na). This is a drug used against epileptic seizures, and also as a prophylactic treatment in traumatic brain injury, which leads to the formation of ROS and the consequent DNA damage. It was found that melatonin (and/or its metabolites) attenuates the genotoxic effects of PHT-Na, and that it can reverse the DNA damage induced by this compound. This can be directly associated with the ROS scavenging activity of melatonin, and led the authors to proposed melatonin as an add-on antioxidant in the treatment of patients needing PHT-Na.

It has been proposed that melatonin may prevent the DNA damage caused by hyperglycemic conditions, by scavenging the excess of ROS derived from it [205]. It was found that the administration of 10 mg/kg melatonin over six weeks has beneficial effects for diabetic rats. Such a treatment decreased OS parameters such as % tail DNA and mean tail moment. There is also evidence that melatonin has protective effects against the genotoxicity induced by cyclophosphamide [206], which is a medical drug used in chemotherapy and to suppress the immune system. Albeit, the exact mechanism of such protection has not been fully elucidated, it was attributed to the antioxidant and FR scavenging capabilities of melatonin.

There are other studies showing that OS-induced DNA damage in human spermatozoa is inhibited by melatonin. Espino et al. [207] demonstrated that melatonin is capable of protecting ejaculated human spermatozoa against apoptosis caused by OD. In this case, in vitro samples treated with melatonin exhibited higher percentage of motile, progressive motile and rapid cells, as well as reduced number of nonviable spermatozoa, compared with the control; at the same time that ^•^NO levels were significantly decreased. Bejarano et al. [208] found that melatonin supplementation, for 90 days, resulted in an increased total antioxidant capacity in the seminal fluid and in a reduction of OD to sperm DNA. This is in line with other reports on the potential role of melatonin as a spermatozoa protector [209]. The beneficial effects of melatonin on sperm quality have been attributed to its role in inhibiting increased ROS levels, which are related to teratozoospermia, sperm malformations and instability of sperm DNA [210]. 

There is also evidence that melatonin inhibits OS-induced DNA damage in mammalian oocytes. It has been recently reported that melatonin significantly decreased intracellular ROS levels, and the associated DNA damage, in aged bovine oocytes [211]. Melatonin was found to reduce ROS levels, and to inhibit 8-oxo-dG production in mice oocytes; thus protecting DNA from OD-induced mutation [212]. 

UV radiation (UVR) is known to induce serious structural and functional alterations in human skin. ROS have been identified as key species in UVR-mediated photo-damage to skin, due to their potential to induce DNA oxidation [213], which yields 8-OH-dG and leads to carcinogenesis [214]. It has been found that pre-incubation with melatonin significantly reduces the amounts of 8-OH-dG positive cells and prevents antioxidative enzyme gene and protein suppression. Based on this findings it was proposed that melatonin plays a crucial role protecting DNA against UVR-induced OD in human skin. It was hypothesized that the radical scavenging activity of melatonin is responsible for reducing ROS levels, albeit indirect ways of action may be involved, i.e., antioxidative enzymes (protected by melatonin) may also contribute to ROS reduction [215].

The 8-OH-dG lesions in DNA are also induced by microcystin, a liver-specific toxin synthesized by *Microcystis aeruginosa*. This can be ameliorated by melatonin in a concentration-dependent manner. To that purpose, melatonin (IC_50_ = 0.55 μM) was found to be significantly more effective than vitamin C or vitamin E (IC_50_ = 31.4, and 36.8 μM, respectively) [216]. It was proposed that melatonin’s protection against microcystin toxicity is caused, at least in part, by its direct ^•^OH scavenging activity. This radical, together with other ROS, has also been held responsible for the l-cysteine-induced mitochondrial DNA (mtDNA) damage in mice brain. Melatonin was able to completely prevent such damage, which was attributed to its capability of scavenging ^•^OH [217]. Similar results were obtained, and similar conclusions drawn, for other chemical agents (kainic acid and potassium cyanide) that induce oxidative damage to mtDNA [218,219,220]. Moreover, melatonin prevents the damage induced by cyanide, kainate, glutathione/Fe^3+^/O_2_ or H_2_O_2_/Fe^2+^ to calf thymus DNA. Based on these findings, together with the knowledge that melatonin is an excellent ^•^OH scavenger, it was proposed that this radical may play a crucial role in the DNA damage induced by those chemicals [221].

Oxidative DNA damage can arise as a consequence of many other factors. Phosphine (PH_3_) is a widely used pesticide that was found to induce, both in vitro and in vivo, brain DNA oxidation in rats, yielding 8-OH-dG. The capability of melatonin to prevent such a damage was explained based on its FR scavenging activity [222]. Naphthalene is another toxic agent. Its toxicity involves enhanced production of FR and DNA fragmentation, among other effects, which lead to cytoxicity. It has been found that two hours pre-treatment of cultured cells with melatonin significantly inhibits naphthalene’s cytoxicity [223]. 

Exposure to microwave (MW) radiation, in vivo, was found to cause DNA single- and double-strand breaks in brain cells; while melatonin treatment (immediately before or after exposure) was found to prevent this damage [224]. This protection was attributed to the FR scavenging activity of melatonin. It has been reported that hyperoxia is another event that leads to increased ROS levels. Exposing bovine cerebral endothelial cells to 95 or 100% oxygen resulted in DNA fragmentation and cell death. Melatonin was found capable of preventing that outcome, in a dose-dependent way, which was explained based on its ROS scavenging activity [225].

Based on the evidence presented in this section, it can be stated that melatonin is a versatile FR scavenger, capable of deactivating a wide variety of these toxic agents. Such capability has been proven to have beneficial effects regarding OD-induced DNA damage. In particular there is ample evidence showing its role in reducing 8-OH-dG levels, and derived lesions. 

There are some related aspects that still need investigation regarding the protective effects of melatonin against OD induced by chemical agents in general, and FR in particular. The possible chemical mechanisms implicated in the capability of melatonin to protect biomolecules against non-radical ROS, as well on reactive nitrogen species (RNS) and reactive sulfur species (RSS) is one of them. As mentioned before, the reacting FR (or oxidant in general) may influence the chemical route contributing the most to the scavenging activity of chemical antioxidants. Understanding such routes in detail is crucial to design adequate strategies to counteract the toxicity of chemical oxidants, and also to know the intermediate products yield in the process. The latter is important in the context of OD because there is a chance that such intermediates are reactive enough to damage some biological targets. That possibility certainly deserves detailed investigations.

### 3.2. Inhibiting Metal-induced Oxidation

As above mentioned, chelation is a chemical means of inhibiting metal-induced oxidation. This particular process is directly involved in the ^•^OH-inactivating ligand (OIL) [226,227] behavior of antioxidants. The protection exerted by OIL species against ^•^OH-induced OD may involve two different ways of action [228]:

(i) sequestering metal ions from reductants, i.e., inhibiting the reduction of metal ions thus their reduced forms are not available for Fenton-like reactions; or
(ii) deactivating ^•^OH after being produced via Fenton-like reactions. In this case the ^•^OH radicals are still formed, but they are rapidly scavenged by the organic ligands in the metal chelates.

**Scheme 3:**
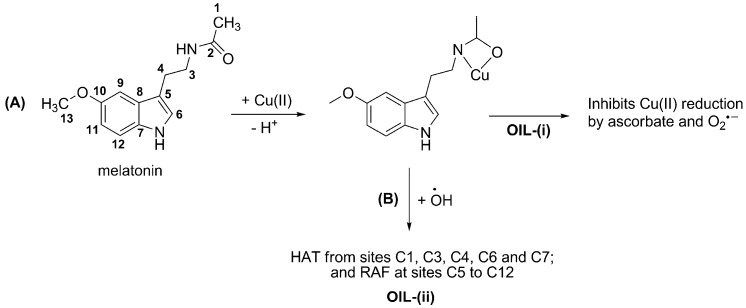
Reaction pathways involved in the ^•^OH-inactivating ligand (OIL) behavior of melatonin. (**A**) previously predicted OIL-(i) activity [18]; (**B**) anticipated OIL-(ii) activity.

To the best of our knowledge, mechanistic insights regarding the OIL behavior of melatonin have been reported only for its role as OIL-(i), when the redox metal is copper [18]. The most likely complex was identified (Scheme 3A) and proved to inhibit oxidation induced by Cu(II)-ascorbate mixtures, as well as the first step of the MC-HWR. Based on the high reactivity of free melatonin towards ^•^OH (described in Section 3.1) it might be anticipated that this molecule can also be efficient as an OIL-(ii) antioxidant (Scheme 3B). However, further investigations on this subject are still needed.

It is known that melatonin is capable of chelating several metal ions including iron, copper, aluminum, lead, cadmium, and zinc [229]. By binding these metals, melatonin retards Fenton reactions, preventing ^•^OH generation. Moreover, melatonin protects DNA against OD induced by Fenton reagents. It over-performs, in this capacity, other antioxidants such as resveratrol, ascorbic acid, and lipoic acid [230]. There is also evidence that melatonin is capable of significantly decreasing the amounts of FR yielded by the interactions of Fe(II), Cu(II), Al(III), Zn(II), and Mn(II) with the amyloid-beta peptide [231]. It has even been suggested that the antioxidant and neuro-protective activities of melatonin may involve removing toxic metals from the central nervous system [46].

In the particular case of iron (Fe), it has been demonstrated that melatonin’s complexes involve Fe(III), although not Fe(II) [229]. This finding led to the proposal that Melatonin removes free Fe(III) from biological environments, thus preventing its reduction to Fe(II), and the associated production of FR. In the presence of iron, d-aminolevulinic acid generates ROS, via Fe-catalyzed oxidation, which leads to the formation of 8-OH-dG lesions [232]. Melatonin inhibits the formation of such lesions, in calf thymus DNA, in a dose-dependent manner [233,234]. To that purpose, melatonin was found to be more efficient than mannitol and Trolox [233]. The inhibitory effects of melatonin on oxidative DNA damage induced by ferric nitrilotriacetate (Fe-NTA), in the rat kidney, have also been investigated. It was found that pre-treatment with melatonin prevents such damage, significantly reducing the levels of 8-OD-dG [235]. It was inferred that the toxicity of Fe-NTA arises from the production of ROS, and that melatonin protection is a consequence of its FR scavenging activity and other antioxidative processes induced by this compound. The DNA strand breaks caused by the exposure of rat lymphocytes to iron ions and 50 Hz magnetic field, simultaneously, was found to be inhibited by melatonin [236]. Such inhibition takes place in a dose-dependent manner, with melatonin 0.5 mM and 1.0 mM leading to 50% and 100% inhibition, respectively.

Copper (Cu) is also an active metal in the context of MC-HWR and/or Fenton reactions. It has been demonstrated that DNA damage caused by mixtures of Cu(II) + H_2_O_2_, at pH 7.4, is greater than that derived from Fe(III) + H_2_O_2_, under the same conditions [237]. It was also found that the damage increases in the presence of ascorbic acid. This can be attributed to the fact that at pH 7.4 the ascorbate anion is the most abundant form of ascorbic acid; thus it can act as a reductant for both Cu(II) and Fe(III). In other words ascorbate promotes de formation of ^•^OH via Fenton reaction. This seems to be confirmed by the finding that 8-OH-dG is the main product yielded in both cases. In addition, the DNA damage was inhibited by metal chelating agents but not by FR scavengers [237]. Therefore, it seems that under the above mentioned conditions the inhibition of OD to DNA is mediated by OIL-(i) behavior, rather than by direct AOC. This is in line with recent theoretical predictions that the oxidative damage induced by copper can be successfully inhibited by melatonin, through its chelating capability [18,19]. There is also experimental evidence that melatonin protects against copper-mediated FR damage [46].

Chromium (Cr) may exist in diverse oxidation states. The Cr(VI)/Cr(V) pair has been reported to mediate Fenton-like reactions, producing ROS that ultimately lead to DNA damage [238]. Melatonin was found to protect DNA from strand break injuries caused by Cr (VI) [239]. Cr(III) can also induce oxidative damage to DNA. In fact it has been reported that Cr(III) is more reactive than Cr(VI) towards DNA, under in vitro conditions [240]. There is abundant evidence that Cr(III)-induced 8-OH-dG lesions can be inhibited by melatonin and other antioxidants [97,230,240,241]. However, melatonin was found to be more efficient for that purpose than ascorbate, Trolox, resveratrol, xanthurenic acid and lipoic acid [97,230,240]. Moreover, melatonin significantly increases the protective effects of ascorbate and lipoic acid against Cr-induced oxidative damage to DNA [230]. The formation of 8-OH-dG has been attributed to Cr(III)-mediated Fenton-type reaction yielding ^•^OH [97]. Thus, the protective effects of melatonin in this context have been proposed as promising for reducing the incidence of Cr-related cancers [97,240]. It has been suggested that the protective effects of melatonin against oxidative damage to DNA induced by chromium can be exerted by (i) scavenging ^•^OH, (ii) directly detoxifying H_2_O_2_, and/or (iii) metal chelation [230].

Lead (Pb) genotoxicity has been attributed to the increase of ROS levels and to the inhibition of DNA repair [242]. The first one is two-fold, at least, and includes direct ROS production as well as depletion of the cellular antioxidant pool [243]. Melatonin was found to significantly attenuate, in vivo, the effects of Pb on DNA repair in rat lymphocytes, albeit its efficiency depends on the administered Pb dose [243]. In addition, it can be inferred that the melatonin’s protection against Pb-induced toxicity is mainly related to its ability of scavenging ROS, since it had only minor effects on the GSH levels.

It has been reported that Ni(II) can induce DNA damage acting as a catalyst for the Fenton reaction, or by disrupting DNA repair systems [232]. Exposure to nickel causes the production of mitochondrial 8-OH-dG and reduces mtDNA content and transcript levels. These damaging effects have been associated with Ni-induced neurotoxicity, and are attenuated by melatonin [244]. This led to the suggestion that melatonin may have pharmacological potential in protecting mtDNA against the adverse effects of nickel in the nervous system.

Cobalt (Co) is also a threat to DNA integrity [245,246]. This metal can cause DNA-protein cross-linking and disruption of the DNA repair system. In addition, it has been proposed that OS may be involved in the Co-induced cytotoxicity and genotoxicity [247]. In the same work, it was found that melatonin lowers tail DNA % and olive tail moment in rat kidney cells exposed to Co nanoparticles. The protective effects of melatonin in this case were attributed to its capability of lowering ROS levels. Considering that Co-induced FR production seems to be influenced by metal chelation and that melatonin can chelate this metal, Romero et al. [232] proposed metal chelation as an alternative route to explain the protection exerted by melatonin against Co toxicity.

The toxicity of mercury (Hg) involves a wide variety of mechanisms, some of which are relevant in the context of this review. Hg has been reported to significantly increase ROS levels and OS, to deplete GSH levels, to damage DNA and to cause failure in the DNA repair machinery [248,249,250]. In particular, Hg-induced genotoxic effects are believed to be mediated by its oxidant behavior, which can lead to DNA damage involving both the purine-pyrimidine bases and the deoxyribose units [249,251]. It has been recently demonstrated that melatonin and vitamin E can both inhibit the Hg-induced genotoxicity, and that their protective effects are potentiated when simultaneously administered [249]. This finding seems to support the hypothesis that melatonin’s protection against the toxic effects of Hg is mediated by its antioxidant capacity [252].

Exposure to arsenic (As) has been reported to cause oxidative DNA damage, as evidenced by the formation of 8-oxo-dG lesions, strand breaks, DNA-protein crosslinks and abnormal DNA methylation, as well as impairment of the DNA repair system [253]. DNA damage induced by arsenite are mediated by ROS, which is in line with observations of depleted GSH levels and the protection provided by antioxidants. Melatonin was found to protect human blood cells, in vitro, against the DNA damage induced by As, which was attributed to the antioxidant potential of melatonin [254]. In addition, it has been reported that methylated As can occur in vivo and lead to DNA oxidation, which is prevented by melatonin and other antioxidants. This finding led to the conclusion that the indirect genotoxic effects of As are mediated by ROS [13].

The protection against metal-catalyzed molecular damage provided by melatonin has been recently, and comprehensively, reviewed [232]. It was proposed that the mechanism involved in the protection exerted by melatonin, in this context, is manifold and involves metal chelation, direct free radical scavenging and promotion of the activity and expression of antioxidant enzymes. It was suggested that the low toxicity of this compound and its ability to easily cross cellular membranes are contributing factors to the efficiency of melatonin for counteracting metal-induced damage to biological molecules, including DNA.

Regardless of the action mechanism, it can be stated that melatonin protects biomolecules in general, and DNA in particular, against metal-induced OD. However, more investigations on the chemical routes involved in such protection are still needed. In particular, mechanistic insights on the OIL-(i) and OIL-(ii) behavior of melatonin would be of great interest. In addition, the relative importance of the possible mechanisms, depending on the involved metal, would be relevant. From a chemical point of view OIL behavior may –arguably- be the most evident way of protecting biomolecules against metal-induced OD. However, metals can lead to OD by routes other than Fenton-like reactions. Such a possibility also deserves further investigation.

### 3.3. Counteracting the Effects of Other OD Triggers

**Scheme 4:**
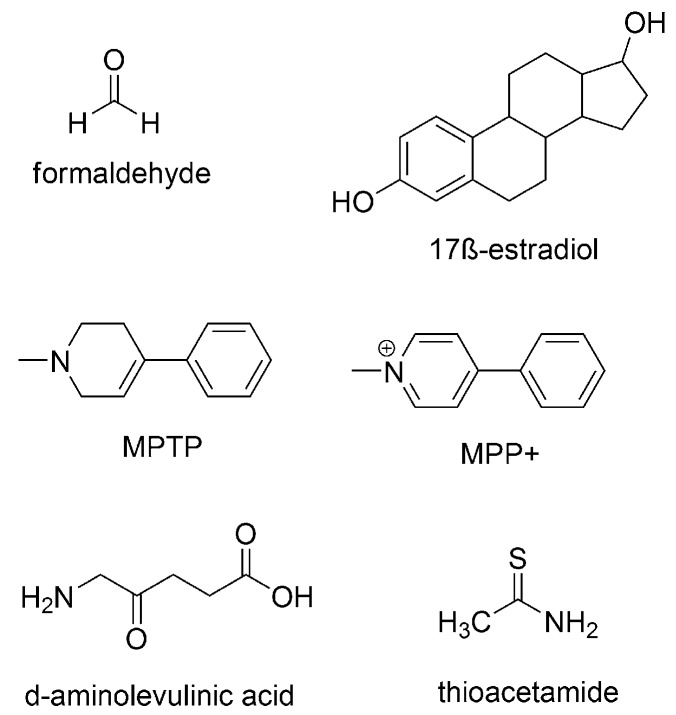
Structures of some chemicals involved in oxidative damage to DNA.

In addition to FR and active redox metals there are other factors that can trigger OD to DNA. A few examples including some chemicals (Scheme 4), and other threatening factors are presented next.

It has been reported that exposure to formaldehyde increases the levels of DNA damage. This compound causes raised levels of ROS and depletion of GSH, among other effects, which ultimately induce morphological changes in tissues [255]. Melatonin can significantly ameliorate formaldehyde’s toxicity. This protection likely involves reduction of ROS levels and DNA damage (in particular 8-OH-dG lesions), a balance of the oxidant/antioxidant status and an inhibition of neutrophil infiltration [255].

17β-Estradiol (E2) has been reported to produce DNA damage, which is reflected in the 8-oxo-dG levels. Thus the toxic effects of this compound might be mediated by OD. Melatonin prevents such a damage in hamster kidneys, which led to the proposal that melatonin is a likely protector from the E2-induced carcinogenesis [100]. 

1-Methyl-4-phenyl-1,2,3,6-tetrahydropyridine (MPTP) is a precursor of 1-methyl-4-phenyl-pyridine ion (MPP^+^). The MPTP/MPP^+^ pair has known neurotoxic effects and may induce Parkinson's disease [256,257,258]. It has been shown that this pair causes OD to mtDNA, as indicated by the presence of 8-oxo-dG [101] that, as previously mentioned, is considered as a biomarker of this kind of damage. In the same study it was demonstrated that melatonin can offer protection against the cell death induced by MPTP/MPP^+^. The protective effects of melatonin, in this context, were attributed to its capability of inhibiting generation of mitochondrial oxygen FR and preventing mitochondrial membrane potential collapse. 

It has been found that potassium bromate (KBrO_3_) induces oxidative DNA damage in the kidney of rats. After treatment with this chemical, the levels of 8-oxo-dG in renal genomic DNA increased by more than 100% [259]. This effect was partially inhibited by melatonin, which was explained by its antioxidant activity.

Exposure to hypochlorous acid (HOCl) can cause DNA strand breaks and modified nucleotides (including oxidation of pyrimidine bases and chlorination of cytosine) in human respiratory tract epithelial cells [260]. Such DNA damage is thought to be involved in HOCl cytotoxicity, together with protein damage manifested as carbonyl formation and oxidation of thiol groups. Melatonin was found to protect cells from the molecular damage caused by HOCl exposure, diminishing its cytotoxic effects [261]. 

Overproduction and accumulation of d-aminolevulinic acid (dALA) can lead to DNA damage and, eventually, to carcinogenesis. Toxic levels of dALA arise as a consequence of acute intermittent porphyria, hereditary tyrosinemia, lead poisoning, and photodynamic therapy. The damaging effects of dALA to DNA have been measured based on the production of 8-OH-dG sites in rat lung and spleen homogenates. Melatonin treatment was found to completely inhibit the increase in 8-OH-dG levels caused by dALA [102]. The hazard posed by dALA to DNA integrity was associated to the formation of ROS. Thus the protective effects of melatonin may arise from its antioxidant activity. 

Thioacetamide induces DNA fragmentation and depletion of GSH levels, among many other effects, leading to hepatic fibrogenesis. Melatonin was found to inhibit such effects by decreasing OS levels and DNA damage [103], at the same time that it provided some additional benefits. 

Currently it is well known that the amyloid beta peptide (βAP) is involved in the Alzheimer’s disease. In fact, it is considered the main neuropathologic marker of this disease. It has been found that exposure to βAP resulted in significant OD to mtDNA [106,262], while addition of melatonin prevents the damage in human neuroblastoma cells [106]. Moreover, it was proposed that melatonin may be better than other antioxidants for treating the Alzheimer’s disease. This proposal was based on some additional appealing properties of melatonin, such as its low toxicity, its ability to cross biological barriers, and its inhibitory effects in βAP aggregation.

Melatonin has also been found to prevent radiation-induced damage to DNA [107,108,109,263,264]. For example, it has been proven to reduce DNA fragmentation in the testes of rats exposed to microwaves, by reducing OS levels [107]. Melatonin inhibits strand breaks in human cells exposed to X-ray radiation [108]. Pre-treatment with melatonin also prevents DNA strand breakage in rat brain [109] and 8-OH-dG lesions in rat liver [264], exposed to ionizing radiation (1000 and 800 cGy, respectively).

Some health disorders may lead to DNA damage as well. Ischemia/reperfusion induces increased levels of 8-OH-dG and thiobarbituric acid reactive substances, which are byproducts of lipid peroxidation. Pre-treatment with melatonin significantly prevents the formation of both markers [110]. There is additional evidence supporting the protective effects exerted by melatonin against ischemia/reperfusion-induced OD to DNA [265,266,267,268].

Intracerebral hemorrhage (ICH) significantly increases OS, leading to DNA damage involving the formation of 8-OH-dG sites, apurinic/apyrimidinic abasic sites and depletion of DNA repair [269]. It has been recently demonstrated that melatonin treatment alleviates ICH-induced DNA damage. It was proposed that the protective effects of melatonin in this context is multifaceted, impacting apoptosis, inflammation, OS levels, DNA damage, brain edema, and mitocondrial membrane permeability [111]. The results from that work led to the suggestion that melatonin is a promising candidate in the treatment of mitochondrial dysfunction and ICH-induced disabilities.

In addition, it has been suggested that the administration of physiological doses of melatonin may help to prevent age-related oxidative DNA damage in the brain [112]. Such a proposal was made based on the finding that increased levels of serum melatonin significantly decrease the 8-OH-dG content and the 8-OH-dG/dG ratios in the brain of adult mice.

Based on the data presented in this section, it can be stated that melatonin is a versatile protector against OD to DNA, since it prevents the damage caused by a wide variety of factors that may threaten DNA’s integrity. However, most of the information gathered so far account for the overall effects of melatonin. On the contrary, little information is currently available on the chemistry associated to the protection provided by melatonin against the toxic effects of chemical species other than FR. Based on the structures of the compounds shown in Scheme 4, it is likely that the mechanisms involved in both the damage and the melatonin protection would strongly depend on the toxic agent. Properly elucidating such reaction mechanisms is an important step in the way of fully understanding chemical induced OD and antioxidant protection. 

### 3.4. Metabolic Derivatives

**Scheme 5:**
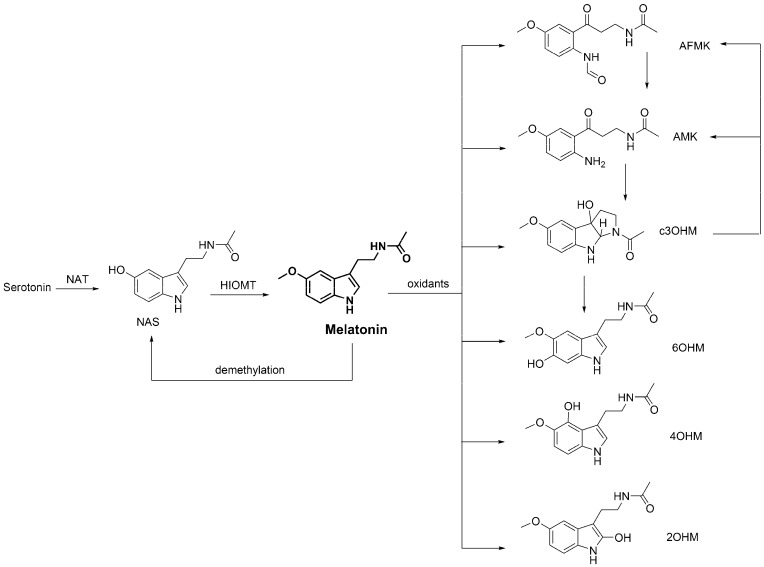
Fragment of the metabolic route of melatonin. NAT = *N*-acetyltransferase, HIOMT = hydroxyindole-*O*-methyl transferase, NAS = *N*-acetylserotonin, AFMK = *N*^1^-acetyl-*N*^2^-formyl-5-methoxykynuramine, AMK = *N*^1^-acetyl-5-methoxykynuramine, c3OHM = cyclic 3-hydroxymelatonin, 6OHM = 6-hydroxymelatonin, 4OHM = 4-hydroxymelatonin, 2OHM = 2-hydroxymelatonin.

Many of the beneficial effects of chemical antioxidants are lost after being metabolized. This is not the case for melatonin, which maintains its protection against OD after being transformed by metabolism or oxidative conditions. There is compelling evidence that melatonin’s metabolites (Scheme 5) are capable of offering protection against oxidative insults. 

*N*-Acetylserotonin (NAS) is not only the direct precursor of melatonin, in the tryptophan pathway, but it can also be reversibly formed from melatonin through demethylation [270]. It has been reported that NAS has neuro-protective effects [271,272] as well as antioxidant and anti-aging activities [273]. NAS can efficiently protect DNA from the OD caused by H_2_O_2_ and Cr(III) [274,275,276] and also from UV-induced damage [277,278]. It also inhibits Cu-induced oxidation [47,48]. 

*N*^1^-Acetyl-*N*^2^-formyl-5-methoxykynuramine (AFMK) is a product of melatonin oxidation, which can involve both enzymatic and non-enzymatic processes [279,280,281,282,283,284,285,286]. AFMK has an excellent ability as a ^•^OH scavenger [287,288,289,290]. It protects against high energy radiation [291] and reduces oxidative DNA damage and lipid peroxidation, preventing neuronal cell injuries caused by H_2_O_2_ [287,288,289,292]. AFMK inhibits the OS induced by Cu(II)-ascorbate mixtures, via Cu(II) chelation [18]. It also protects DNA from UV-induced damage [277,278,289] and from the oxidation caused by Fenton reagents [229] or derived from exposure to mixtures of 5-aminolevulinic acid + Fe(II) [234].

*N*^1^-Acetyl-5-methoxykynuramine (AMK) is formed by deformylation of AFMK [16,51,192,293]. There are several reports showing that AMK is a good and versatile free radical scavenger. It was found to be capable of deactivating a wide diversity of ROS [34,294,295], RNS [35,36,296,297,298] and other oxidants [34,39,40]. There is evidence that AMK also reduces Cr(III)-induced 8-OH-dG lesions in isolated calf thymus DNA [97].

Cyclic 3-hydroxymelatonin (c3OHM) is believed to be a non-enzymatic product of melatonin, which is yielded from the reactions of melatonin with oxidants, particularly ^•^OH [37]. Albeit it was once thought to be a biomarker of OS [299], currently c3OHM is known to be not an end-product, which can be further metabolized into AFM and AMK [52,300]. It is also known that such a transformation is mediated by FR [28]. c3OHM efficiently scavenges ^•^OH [301], ABTS^•+^ (2,2′-azino-bis(3-ethylbenzthiazoline-6-sulphonic acid)) [300] and peroxyl radicals [27]. It is also capable of chelating Cu(II), preventing its reduction and the consequent ^•^OH production through Fenton-like reactions [18]. This theoretical prediction is supported by experimental evidence demonstrating that c3OHM inhibits oxidative DNA damage and 8-OH-dG lesions, induced by Fenton reagents, under in vitro conditions [301]. 

6-Hydroxymelatonin (6OHM) has been identified as a major melatonin metabolite in the human skin [154,278,302]. It is also a product yielded by the reaction of melatonin with ^•^OH, in Fenton-type generating systems [282] and by the UV-induced metabolism of melatonin in keratinocytes and cell-free systems [285]. It has been reported that 6OHM can reduce neurotoxicity induced by quinolinic-acid, due to its capability of scavenging ^1^O_2_ and O_2_^•−^ [303]. It also lowers Fe(II)-induced neurotoxicity [44] and inhibits the OD induced by this metal [43,44], UV radiation [295], thiobarbituric acid [304] and cyanide [305]. In addition, it has been proposed to inhibit OS induced by Cu(II)-ascorbate mixtures and ^•^OH production [18]. 6OHM also protects DNA damage induced by Fenton reagents [301] and UV radiation [277,306]. The latter is believed to take place by enhancing the DNA repair in exposed melanocytes. 

4-Hydroxymelatonin (4OHM) and 2-hydroxymelatonin (2OHM) are generated during the UV-induced metabolism of melatonin [285]. In addition, they can be produced during the oxidation of melatonin through chemical routes. For example, 2OHM is formed due to the reactions of melatonin with hypochlorous acid [307], oxoferryl hemoglobin [308] and ^•^OH [282]. The latter also produces 4OHM. To the best of our knowledge, there are no previous studies indicating whether, or not, 2OHM and 4OHM can play a protective role against OS-induced damage to DNA. The information regarding their antioxidant capacity is also very scarce. It has been recently proposed, based on theoretical calculations, that 4OHM may play an important role on the protective effects of melatonin against OS. On the contrary, the effects of 2OHM in this context were predicted to be only minor. The antioxidant activity of these two compounds, as well as their potential role in protecting biomolecules against OD certainly deserves further investigation.

AFMK, AMK, c3OHM, 6OHM, 4OHM and 2OHM are all products yielded by the oxidation of melatonin. Since most of them also have antioxidant properties, the protection exerted by melatonin against OD is a continuous process in which multiple oxidative offenders can be deactivated. Moreover, part of the antioxidant protection exerted by melatonin may be attributed to its metabolites [173]. In this regard melatonin is a particularly virtuous molecule. Most of the antioxidants, when metabolized, are transformed into species that do not necessarily offer protection against OD. Melatonin, on the other hand, is one of the few known antioxidants that does not loose AOC during metabolism. 

### 3.5. Other Protection Mechanisms

This aspect would be only briefly addressed here since it is not within the main focus of this review. However, it seems important to emphasize the fact that there are several mechanisms (other than those more extensively reviewed here) that may significantly contribute to the indirect antioxidant activity of melatonin. A few examples of the evidence gathered so far, regarding some of them, are provided here. 

#### 3.5.1. Activating Antioxidative Enzymes

The protective effects of melatonin against radiation-induced DNA damage has been partially attributed to its indirect antioxidant activity. It has been proposed that its protection against UV-induced damage to human skin involves preventing the depletion of the antioxidative enzymes catalase, glutathione peroxidase and superoxide dismutase (SOD) [215,309]. 

The fact that melatonin lowers the neurotoxic effects induced by 6-hydroxydopamine [310] has been attributed to both its free radical scavenging activity and its role in adjusting the activity of Mn-SOD and Cu/Zn SOD [15]. As in many other works, mentioned here, it seems that in this case the antioxidative protection exerted by melatonin involves multiple ways of action.

Exposure to bisphenol A can lead to DNA damage, which is believed to be mediated by increased OS levels and is accompanied by depletion of SOD activity [104]. The capability of melatonin to prevent such alterations may then be attributed to both its direct antioxidant activity and its involvement in activating SOD. The role of melatonin in preventing OD and modulating SOD has also been related to the protective effects of this molecule against DNA damage induced by carbon-ion beam irradiation [311].

There is also evidence that melatonin and its metabolites (6OHM, AFMK and NAS) protect melanocytes from UVB-induced DNA damage and OS through activation of nuclear erythroid 2-related factor 2 (Nrf2) and its target enzymes [277]. Induction of Nrf2 has also been associated with the effects of melatonin in reducing oxidative and nitrosative DNA damage [312]. In fact, numerous investigations have shown that melatonin activates phase-2 antioxidative enzymes, via the Nrf2 pathway, including heme oxygenase-1 (HO-1), γ-glutamylcysteine synthetase (γ-GSC), nicotinamide adenine dinucleotide phosphate (NADPH): quinone dehydrogenase-1 (NQO1) and SOD [313,314,315,316,317,318,319,320,321,322,323,324,325,326,327,328,329,330,331,332,333].

There are many other reports in the literature documenting the role that antioxidant enzymes play in the protective effects exhibited by melatonin against OD [170,194,334,335,336,337,338]. In addition, it seems that melatonin concentrations influence the relative importance of direct FR scavenging versus indirect actions involving activation of enzymatic pathways [339]. Apparently, high concentrations (~1 mmol) favors the first, while low concentrations (~100 nm) promotes the enzymatic removal of ROS. 

#### 3.5.2. Inhibiting Pro-oxidative Enzymes 

It has been reported that the activity of xanthine oxidase (XO), an enzyme that generates ROS, increases after exposure to MW radiation [107,340,341,342,343]. At the same time, melatonin has protective effects against DNA fragmentation, induced by this kind of radiation. Thus, the beneficial effects of melatonin in this context has been attributed, at least partially, to the capability of melatonin for lowering the OS produced by XO [107]. It is not clear, though, if in this case melatonin acts only as a ROS scavenger or if it actually inhibits XO activity [342]. However, in a different study conducted on kidney tissue after ischemia and reperfusion [267], it was proposed that melatonin neutralizes the products of the XO + O_2_ reaction, rather than directly suppressing the XO activity. Therefore, it seems likely that the protection offered by melatonin against MW radiation is also mediated by its capability of scavenging ROS.

The protective effect of melatonin against *Opisthorchis viverrini*-induced oxidative and nitrosative stress and liver injury were investigated in hamsters [312]. It was found that the formation of DNA lesions, namely 8-oxo-dG and 8-nitroguanine, is inhibited by melatonin. This protection was attributed to the inhibitory effects of melatonin on the mtRNA expression of oxidant generating genes, including inducible nitric oxide synthase, nuclear factor-kappa B and cyclooxygenase-2. Although, at the same time melatonin seems to increase the expression of antioxidant genes Nrf2 and Mn-SOD.

#### 3.5.3. Boosting DNA Repair Machinery

It has been reported that pre-treatment with melatonin (100 mg/kg) protects rats exposed to whole-body X-ray radiation by modulating 8-oxoguanine glycosylase1 (Ogg1), apurinic/apyrimidinic endonuclease (Apex1) and X-ray repair cross-complementing group 1 (Xrcc1) gene expression in peripheral blood cells [263]. These genes are among the most important ones for dealing with FR-induced DNA damage, in the base excision repair (BER) pathway. Ogg1 is involved in the removal of 8-oxo-dG DNA lesions [344], Apex1 mediates the repair of abasic sites [345] and Xrcc1 is required for repairing strand breaks [346]. Thus the finding that melatonin modulate these genes has a direct impact on reducing DNA damage. Moreover, based on the finding that melatonin may increase the normal tissue tolerance to radiation, by enhancing DNA repair, it was proposed that this molecule might be used to downgrade radiation toxicity in patients undergoing cancer radiotherapy [263].

Also relevant for cancer patients are the finding that melatonin reduces the DNA damage induced by cyclophosphamide [347], which is an anti-tumor agent currently used in clinical practice. The protective effect of melatonin in this case was attributed to its capacity to up-regulate the XPF expression, which is involved in the DNA nucleotide excision repair machinery. In view of that, melatonin administration was proposed as a co-treatment during chemotherapy.

Pre-treatment with melatonin was reported to increase DNA repair capacity in breast and colon cancer cells exposed to the mutagen methyl methanesulfonate [105]. In the same study the genome-wide gene expression was examined and it was found that melatonin leads to altered expression of many of the investigated genes. This led to the suggestion that melatonin may enhance DNA repair capacity by affecting several key genes involved in DNA damage responsive pathways.

The effects of melatonin on DNA double-strand breaks caused by ionizing radiation were investigated using 108 male Wistar rats. The results from this investigation showed that the administration of melatonin (100 mg/kg), 8 and 24 h before exposure, significantly promotes DNA repair in non-homologous end joining pathways by increasing the expression of genes Ku70 and Xrcc4 [348].

It is very interesting, in this context, that melatonin was found to reduce the time of DNA repair by one-half, although it does not seem to increase the activity of the base-excision repair glycosylases [285]. It was then proposed that the melatonin’s ability to accelerate the DNA repair might involve other routes including: (i) chemical inactivation of H_2_O_2_; (ii) stimulation of DNA repair pathways other than BER; and/or (iii) interaction with BER-related enzymes other than glycosylases, or with their cofactors.

There is another interesting proposal regarding the relationship between melatonin’s anticancer activity and its role preserving DNA integrity. Santoro et al. [349] proposed that melatonin-induced enhancement of the DNA repair machinery might (i) prevent carcinogenesis in healthy individuals; (ii) inhibit mutations in pre-cancerous lesions; (iii) reduce the risk that cancer cells mutate into more aggressive phenotypes; and (iv) limit the side effects of anticancer therapy in healthy tissues.

The role of melatonin in modulating DNA damage response and repair pathways has been recently, and thoroughly, reviewed [171]. Thus the interested reader is referred to that work for further details on this topic.

## 4. Concluding Remarks

The beneficial effects of melatonin as an antioxidant have been profusely documented in the literature. In the particular case of its protection against oxidative DNA damage, the evidence gathered so far clearly indicates that melatonin is an astonishingly versatile molecule in this context. It can offer both direct and indirect protection against a wide variety of damaging agents and through multiple pathways, which may (or may not) take place simultaneously.

The direct antioxidative protection of melatonin is evidenced by its efficiency for scavenging free radicals, which are frequent triggers of oxidative damage to DNA. Melatonin has been proven to deactive several of these species including hydroxyl, alkoxy and peroxy radicals. Melatonin can also deactive non-radical reactive oxygen, and nitrogen, species. Such protection is evidenced by the melatonin effects in reducing 8-OH-dG, and associated DNA lesions.

The indirect antioxidative protection of melatonin involves many ways of action. It can protect DNA against metal-induced damage. Such protection seems to be quite general, since melatonin has been proven to inhibit the deleterious effects of a wide variety of metals including Fe, Cu, Cr, Pb, Ni, Co, Hg and As. Such protection itself may involve different mechanisms. One of them is mediated by the chelating capabilities of melatonin, which leads to the prevention of Fenton-related ^•^OH generation. 

There is also evidence supporting the protective effects of melatonin against other triggers of oxidative DNA damage. They include chemical agents such as formaldehyde, 17β-estradiol, the MPTP/MPP^+^ pair, potassium bromate, D-aminolevulinic acid and thioacetamide; as well as radiation and some health disorders.

In addition, melatonin’s metabolites (including AFMK, AMK, c3OHM, 6OHM and 4OHM) also have antioxidant properties. Thus, the protection exerted by melatonin against oxidative damage to DNA is a continuous, and virtuous, process in which multiple oxidative offenders can be deactivated. Moreover, part of the antioxidant protection exerted by melatonin may be attributed to its metabolites

Other indirect pathways contributing to melatonin’s protective effects against oxidative damage to DNA involve activating antioxidative enzymes, inhibiting pro-oxidative enzymes and boosting the DNA repair machinery. The rather unique capability of melatonin to exhibit multiple ways of actions, against diverse threatening factors, together with its low toxicity and its ability to cross biological barriers, are all significant to its efficiency for preventing oxidative damage to DNA.
